# Auxin-Cytokinin Cross Talk in Somatic Embryogenesis of *Coffea canephora*

**DOI:** 10.3390/plants11152013

**Published:** 2022-08-02

**Authors:** Johny R. Avilez-Montalvo, Ana O. Quintana-Escobar, Hugo A. Méndez-Hernández, Víctor Aguilar-Hernández, Ligia Brito-Argáez, Rosa M. Galaz-Ávalos, Miguel A. Uc-Chuc, Víctor M. Loyola-Vargas

**Affiliations:** 1 Unidad de Bioquímica y Biología Molecular de Plantas, Centro de Investigación Científica de Yucatán, Calle 43, No. 130 × 32 y 34, Mérida 97205, Mexico; johnyavilez@gmail.com (J.R.A.-M.); ana.quintana@estudiantes.cicy.mx (A.O.Q.-E.); hugo.mendez@cicy.mx (H.A.M.-H.); lbrito@cicy.mx (L.B.-A.); gaar@cicy.mx (R.M.G.-Á.); ma.uc@outlook.com (M.A.U.-C.); 2 Catedrático CONACYT, Unidad de Bioquímica y Biología Molecular de Plantas, Centro de Investigación Científica de Yucatán, Mérida 97205, Mexico; victor.aguilar@cicy.mx

**Keywords:** auxins, cytokinins, somatic embryogenesis, *Coffea canephora*, cellular differentiation

## Abstract

Cytokinins (CK) are plant growth regulators involved in multiple physiological processes in plants. One less studied aspect is CK homeostasis (HM). The primary genes related to HM are involved in biosynthesis (IPT), degradation (CKX), and signaling (ARR). This paper demonstrates the effect of auxin (Aux) and CK and their cross talk in a *Coffea canephora* embryogenic system. The transcriptome and RT-qPCR suggest that Aux in pre-treatment represses biosynthesis, degradation, and signal CK genes. However, in the induction, there is an increase of genes implicated in the CK perception/signal, indicating perhaps, as in other species, Aux is repressing CK, and CK are inducing per se genes involved in its HM. This is reflected in the endogenous concentration of CK; pharmacology experiments helped study the effect of each plant growth regulator in our SE system. We conclude that the Aux–CK balance is crucial to directing somatic embryogenesis in *C. canephora*.

## 1. Introduction

More than 60 years ago, Miller et al. [1,2] discovered kinetin (6-furfurylaminopurine or *N*^6^-furfuryladenine) in autoclaved herring sperm DNA. Kinetin is a potent promotor of the proliferation of cultured tobacco calli from pith cells and belongs to the group of plant growth regulators (PGR) named cytokinins (CK). The cross talk between auxins (Aux) and CK, two key PGR, was found soon after the discovery of CK [3]. The relationship between Aux and CK, together with other plant growth regulators, promotes and maintains the fine-tuned regulatory functions that control plant growth and development [4,5,6,7]. This interaction happens at different levels [8,9].

At the molecular level, it has been shown that at the shoot apical meristem (SAM), Aux and CK act on the promotors of two negative regulators of CK signaling, the A-Type *ARABIDOPSIS RESPONSE REGULATOR* (*ARR*) genes, *ARR7/ARR15*. CK in the SAM induce the expression of both genes, and the Aux negative effect is mediated by the AUXIN RESPONSE FACTOR5/MONOPTEROS (MP) transcription factor [10]. This mechanism provides a form through which Aux can provide input to regulate the sensitivity of a subdomain of the SAM to CK [11]. The CK receptors AHK2/AHK3 are mainly responsible for the transduction of the CK signal to B-Type response regulators, in particular to the transcription factors ARR1/ARR10/ARR12 [12]. The AHK4 CK’ receptor mediates induction of *WUS* specifically in the disorganized primordia, leading to the acquisition of shoot identity and activation of the downstream developmental cascade, allowing de novo shoot formation [13].

CK regulate local Aux metabolism [14,15,16], modulate the Aux pool by disrupting its transport [17,18,19], and regulate the expression of PIN-FORMED (PIN) Aux transporters through transcription factors downstream of CK perception, the CYTOKININ RESPONSE FACTORS [20]. CK also modulate signaling through the activation of ARABIDOPSIS RESPONSE REGULATOR 7 (ARR7) and ARR15 [9,21].

CK induce SHY2 (a member of the auxin-induced Aux/IAA family) in the transition zone of the roots by activating transcription by ARR Type B [22]; in turn, SHY2 increases the expression of ISOPENTENYLTRANSFERASE 5 (IPT5) and increases CK biosynthesis. SHY2 reduces expression of the Aux efflux carriers PIN3/PIN4/PIN7 [19,23,24], which depend directly on Aux concentration. The switch-off of the SHY2 signal is carried out by Aux [25,26]. In this way, SHY2 acts as the central control of the mechanism that regulates the homeostasis of CK and Aux. CK act as a second messenger for Aux. In *Pisum sativum*, the indole-3-acetic acid (IAA) exported by the branches is controlled by the CK present in the xylem, which in turn is feedback-regulated by apical Aux [27].

To maintain cambial activity, the transcription factor AUXIN RESPONSE FACTOR 5 (ARF5) is phosphorylated by BIN2-LIKE 1 (BIL1), a glycogen synthase kinase 3. This phosphorylation upregulates the negative regulators of CK that signal ARR7 and ARR15, suggesting that BIL1 is a critical mediator between peptide and Aux–CK signaling for the maintenance of cambial activity [28].

Aux regulates CK biosynthesis and signaling. It also mediates the negative control of the biosynthesis of CK by suppressing the expression of the IPT genes in *A. thaliana* [29] and *P. sativum* [30]. Muller et al. [31] demonstrated this cross talk in *A. thaliana* gynoecium, in which CK positively regulates *YUCCA1/YUCCA4* (*YUC1/YUC4*) as well as *PIN7*, but represses *PIN3* expression, promoting Aux accumulation in the apex. Aux also represses CK signaling by triggering *ARABIDOPSIS HISTIDINE PHOSPHOTRANSFER PROTEIN6* (*AHP6*) [32]. However, in Arabidopsis roots, Aux activates *IPT5/IPT7* genes [33]. This Aux/CK cross talk is extended to fruit development of the tomato, where CK modulate Aux biosynthesis and/or polar auxin transport to prevent abscission of ovaries [34].

Recently, the discovery of an indole-3-butyric acid (IBA) transporter, the TRANSPORTER OF IBA1 (TOB1), that blocks the formation of the lateral roots, adds a new layer of complexity to the already complex interaction between Aux and CK [35]. This transporter is induced transcriptionally by CK. IBA can be considered a precursor in the biosynthesis of IAA or an auxin by itself. However, because CK modify the IBA transport and produce a physiological change, this finding clarifies a role for IBA that was previously unknown.

The addition of exogenous Aux and/or CK is central to the induction of somatic embryogenesis (SE). However, its effect on the status of the endogenous PGR and the molecular context for its biosynthesis during SE is still elusive [36]. The elucidation of the interaction between Aux and CK is vital to our understanding, as the balance between Aux and CK is so important to the start of the SE process.

In changing the Aux/CK ratio, Skoog and Miller [3] were able to influence the development of roots and/or shoots from calli tissues. Since then, the variation in this ratio has been used to induce the differentiation process in explants of hundreds of different species. One of these processes is SE [37].

In *Corylus avellana*, the endogenous *N*^6^-isopentenyladenine (iP) type/zeatin (Z) type CK ratio is a good index of the embryogenic competence of explants [38]. The establishment of SAM and apical root meristem (RAM) are critical steps for SE [39]. During the zygotic embryogenesis, the first CK signal appears in the hypophysis during the 16-cell stage [40]. The hypophysis has an asymmetric division that generates two cells that determine the root stem cell niche: the upper cells that generate the quiescent center (this group of cells keeps the signal of the CK and has a low response to Aux) and the lower cells that give rise to columella cells [41]. It has been demonstrated that Aux activates the transcription of gene-negative regulators *ARR7/ARR15* (feedback repressors of CK signaling). As a result, CK production is reduced, and Aux production increases, enabling the establishment of the root pole from the embryo proper [32,40].

Su et al. [42] showed that the early expression of the *WUS-RELATED HOMEOBOX 5* (*WOX5*) and *WUSCHEL* genes could be used as an early marker of embryonic callus. Their correct expression is essential for RAM and SAM initiation and embryonic shoot–root axis establishment [42]. CK response signals are detected in specific regions correlated with induced *WOX5* expression and subsequent SE formation. On the other hand, the overexpression of *ARR7/ARR15* disturbs RAM initiation and SE induction [42]. These results show the close relationship between Aux and CK and how these two PGR regulate the formation of the shoot–root axis during SE.

The induction of SE is dependent on the initial presence of Aux [43]. However, Aux must be removed after the initial induction in most cases. In some circumstances, the SE process is achieved without the addition of PGR. In other cases, a combination of PGR or CK alone must be added to the culture medium. In the case of *Coffea* spp. more than 41% of the protocols used for the induction of SE use CK alone, 45.8% use a combination of CK with Aux, and 12.6% use CK in combination with another PGR [44].

Endogenous IAA increases in response to the presence of an exogenous Aux in the culture medium, as well as the expression of some genes related to its biosynthesis [45]. However, we do not know if the endogenous CK are playing any role during the induction of the SE in *Coffea canephora*. This work aimed to determine the dynamics of the endogenous CK during the induction of SE in *C. canephora*. The CK/Aux ratio changed from very low during the pretreatment to very high during the first days of the induction of SE, suggesting the crucial importance of CK and Aux during the SE of *C. canephora*.

## 2. Results

### 2.1. Phylogenetic Analysis

To understand the evolutionary relationship of proteins related to CK homeostasis among *C. canephora* and other plant species, three unrooted phylogenetic trees were constructed for IPT, CYTOKININ DEHYDROGENASE/OXIDASE (CKX), and ARR from *Arabidopsis thaliana*, *Brassica napus*, *Capsicum annuum*, *Citrus sinensis*, *Daucus carota*, *Fragaria vesca*, *Ipomoea triloba*, *Jatropha curcas*, *Nicotiana attenuate*, *Oryza sativa* Japonica, *Phaseolus vulgaris*, *Pisum sativum*, *Prunus dulcis*, *Solanum lycopersicum*, *Theobroma cacao*, and *Zea mays*. We found 4 CcIPT, 2 CcDAMPP, 5 CcCKX, and 25 CcARRs in a search of *C. canephora*’s genome. It was found that proteins related to CK homeostasis were presented in all sub-families, except in the monocot cluster of rice and maize.

**Figure 1:**
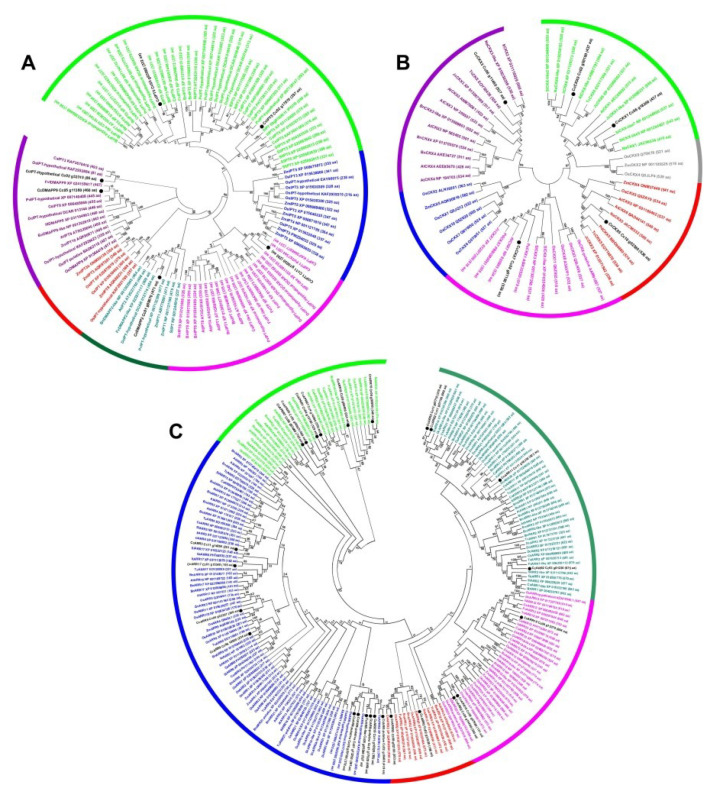
Phylogenetic trees of the CK homeostasis proteins from Coffea canephora Cc, Arabidopsis thaliana At, Brassica napus Bn, Capsicum annuum Ca, Citrus sinensis Cs, Daucus carota Dc, Fragaria vesca Fv, Ipomoea triloba It, Jatropha curcas Jc, Nicotiana attenuate Na, Oryza sativa Japonica Os, Phaseolus vulgaris Pv, Pisum sativum Ps, Prunus dulcis Pd, Solanum lycopersicum Sl, Theobroma cacao Tc and Zea mays Zm. The neighbor-joining method of the MEGA 7.0 program was used to construct the phylogenetic trees; the bootstrap was 1000 replicates: (**A**) the phylogenetic tree of IPT proteins; (**B**) the phylogenetic tree of CKX proteins; (**C**) the phylogenetic tree of ARR proteins. The different colors indicate different groups of proteins. The black letters and black circles show the proteins of *C. canephora*, and the length of amino acids is indicated in parentheses.

The phylogenetic tree of the IPT protein family is clumped into six different groups, with IPT3-5-7 being the big group and the small group being made up of the monocots IPT3-8 (rice and maize) (Figure 1A). The CcIPT1-Cc11_g10030 protein has 38% and 50% homology to CaIPT1 (accession: PHT67344) and to IpIPT-like (accession: XP_031100755), respectively; while CcDMAPP2 shares 62% similarity with DcIPT-Hypothetical (accession: DCAR_012174) (Figure 1A). The proteins CcIPT5-Cc02_g17870 and CcIPT5-Cc03_g03080 are located in the big group and were shown to have 23% and 58% homology to PvIPT-Hypothetical (accession: XP_007139147) and ItIPT5 (accession: XP_031092031), respectively. The CcIPT-Hypothetical-Cc02_g32310 protein has 80% homology to the OsIPT-hypothetical protein (accession: KAF2932884), while only 18% homology between CcDMAPP9-Cc05_g11380 and FvDMAPP9 (accession: XP_024159617).

On the other hand, the phylogenetic tree of the CKX protein family is composed of six different groups (Figure 1B). The first group comprises the CKX1-6-9 and CKX-like2-7 proteins; the proteins CcCKX1-Cc06_g18200 and CcCKX9-Cc02_g30100 are located in this group and have 65% and 94% homology to SlCKX-like7 (accession: NP_001244908) and ItCKX6 (accession: XP_031129313), respectively. Monocots (rice and corn) were grouped into two separate groups; the group formed by the CKX2-4-9 proteins and by the CKX1-2-5-6-7-10 proteins. The CcCKX3-Cc06_g11480 protein has a similarity ratio of 72% and 95% to TcCKX (accession: EOY30438) and NaCKX3 (accession: XP_019230359), respectively, while CcCKX5-Cc10_g02380 is 39% homologous to NaCKX5 (accession: JA96532), and CcCKX7-Cc08_g01180 is 42% homologous to AtCKX7 (accession: NP_850863).

The ARRs represent the largest protein family. The phylogenetic tree produced five different groups (Figure 1C). Interestingly, we found 100% homology between the proteins CcAPRR1-Cc02_g00820 and ItAPRR1 (access: XP_031104980). Furthermore, the CcARR17-Cc01_g15340 protein is 89% homologous to ItARR17 (access: XP_031113979), while CcARR-putative-Cc06_g20160 has 99% homology to OsARR-Hypothetical (access: EAY77400). Several gene paralogs encode for CcARR share homology with the other plant species analyzed in this study (Figure 1C).

### 2.2. Transcriptomic Analysis

**Figure 2:**
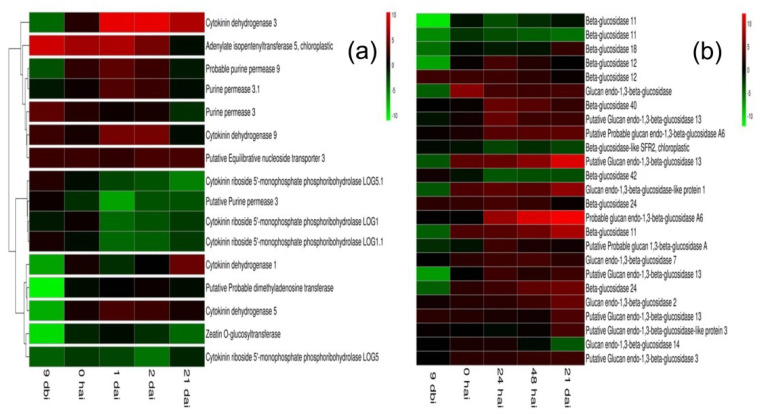
Expression profiles of genes involved in CK metabolism. The heatmap shows changes in transcript levels in different sampling points (-14 days vs. -9, 0, 1, 2, and 21 days). Gene names were annotated using Ugene and Blast2GO software: (**a**) members of CK homoestasis; (**b**) members of the β-glucosidase gene family and change in expression during SE of *C. canephora*. Hierarchical clustering was used to group genes with similar expression profiles. The red represents upregulated genes, and the green represents downregulated genes (log2 fold-change values).

We used the transcriptome information elucidated in our laboratory. This transcriptome includes different points of the embryogenic process of *C. canephora* (14 dbi, 9 dbi, 0 dbi, 1 dai, 2 dai, and 21 dai) [46]. We analyzed the expression of CK genes involved in biosynthesis, transport, degradation, and conjugation (Figure 2). The gene *CcIPT5*, which codes for a key enzyme in CK biosynthesis [47], is induced during the pre-treatment for and the first two days after the induction of SE. The expression of a *tRNA-CcDMAPP* was not induced in the process of SE. The genes codifying for the CYTOKININ RIBOSIDE 5′-MONOPHOSPHATE PHOSPHORIBOHYDROLASE 5.1 (LOG5.1; EC 3.2.2.n1), and the genes *LOG1*, *LOG1.1*, and *LOG5*, decrease sharply after the induction of SE (Figure 2a).

The β-glucosidase (β-Glu; EC 3.2.1.21) cleaves the β-glucosidic linkages of *O*-glucosides of CK into the active form [48,49,50]. From all the *β-Glu* genes expressed during the induction of SE in *C. canephora*, the *β-Glu 11* and *β-Glu 24* genes present a higher level of expression. However, the more highly expressed genes are those from the genes putative glucan endo-1,3-β-glucosidase 13 and probable glucan endo-1,3-β-glucosidase A6 (Figure 2b).

The homeostasis of CK is a highly regulated process. The initial irreversible CK degradation is performed by the family of CKX enzymes [51,52,53]. Of the five CKX genes present in the genome of *C. canephora*, four are expressed during the induction of SE (Figure 2a). The gene *CKX3* is expressed from the beginning of the induction of SE and increases sharply until 21 days after the induction of SE, when the first globular structures appear. The *CKX1*, *CKX5*, and *CKX9* genes also increase their expression level, but not to the same extent as *CKX3* (Figure 2a).

Other genes of the homeostasis of CK analyzed were those involved in their transport and conjugation. CK use the vascular system of the plant for their transport. CK are transported across the plasma membrane. The proteins responsible for this transport are the purine permeases (PUP) [54,55,56,57]. The *C. canephora* genome has five *PUP* genes; the information provided by the transcriptome shows the expression of *PUP3*, *PUP3.1*, *PUP9*, and a putative *PUP3* (Figure 2a). The first three genes did not change their expression during the study period. The putative *PUP3* decreased its expression from the beginning of the induction of SE until the end of the study.

Another important family of genes for CK transport is that of the EQUILIBRATIVE NUCLEOSIDE TRANSPORTERS (ENT) [58]. These transporters mediate the selective translocation of CK nucleosides, which are present in significant proportion in the xylem and phloem [59,60,61]. In *A. thaliana*, the *ENT* family consists of eight members (*AtENT1*-*AtENT8*) [62]. There are four *ENT* genes in *O. sativa* (*OsENT1*-*OsENT4*), where *OsENT2* is expressed in the vascular bundle of leaf and the phloem and takes preference over iPR and tZR [63]. There are two *ENT* genes in the *C. canephora* genome (*CcENT3* and *CcENT4*). However, the transcriptomic analysis shows that only a putative *CcENT3* is expressed throughout the SE process (Figure 2a).

The CK signaling system is carried out using a mechanism known as phosphorelay [64], where the ARABIDOPSIS HISTIDINE KINASE (AHK) and ARABIDOPSIS HISTIDINE-CONTAINING PHOSPHOTRANSFER PROTEIN (AHP) has a significant role in the transfer of the phosphoryl group to the nucleus to activate the *ARR* Type-B and Type-A genes. The *ARR* Type-B gene regulates the transcription of CK-related genes positively, while the *ARR* Type-A gene regulates the *ARR* Type-B genes negatively, regulating CK homeostasis indirectly [11,65].

The gene expression analysis of CK perception/signal yielded interesting results. The *C. canephora* genome contains five *AHK* genes (*CcAHK*) and three *AHP* genes (*CcAHP*) (Figure S1). In the transcriptomic analysis we made, *CcAHK5* and *CcCKI1* showed a differential expression in the SE process (Figure S1). *CcAHK5* was suddenly suppressed during the early days of pre-treatment, while later in the induction it was suppressed in the early hours and increased on the second day until day 21. *CKI1* (*CK INDEPENDENT 1*) is a homolog of the *AHK* genes in Arabidopsis [66,67]. The genome of *C. canephora* has one *CKI1*, which was repressed in the early days of pre-treatment and the first days of the induction of SE; however, during the subsequent days of induction of SE, the expression of *CKI1* increased (Figure S1). The transcriptome analysis shows that only three *AHP* genes (*CcAHP*) are expressed. *CcAHP1* and *CcAHP4.1* were repressed on day -9 during the pre-treatment. Their expression increased during the induction of SE, mainly on the first day of induction, and in the case of *CcAHP1*, its expression was constitutive. *AHP4* presents a different expression pattern, being repressed throughout the process of SE (Figure S1).

The transcriptome of *C. canephora* presents seven *ARR* genes. Three genes of *ARR* Type A (*CcARR3,* 9, and 17) increased their expression during the induction of SE (Figure S1). Transcriptome data showed that *ARR2* (*CcARRR2* Type B) was repressed in all SE processes, *ARR10* (*CcARR10* Type B) was slightly induced in the early days of preconditioning repressed during the induction. Two *APRR* genes (1 and 5) are repressed during all the SE processes, mainly in the induction stage.

### 2.3. Real-Time Quantitative PCR (RT-qPCR) Analysis

**Figure 3:**
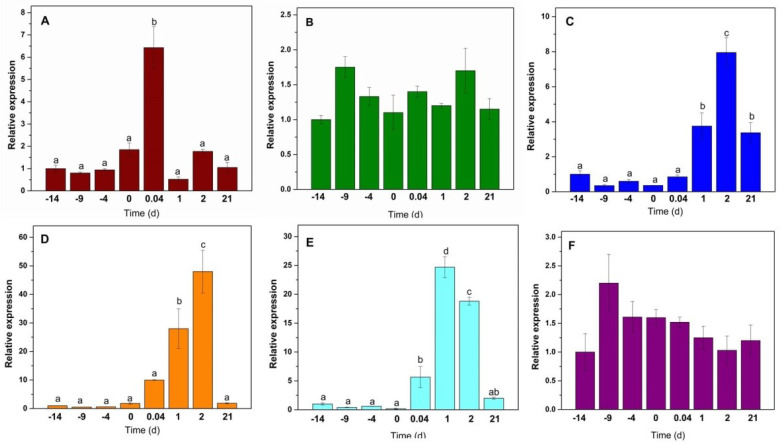
Expression profiles of selected CK biosynthesis and signal gene members in *Coffea canephora*. *Y*-axis values are relative expression (2^−ΔΔCT^ method) averaged from 3 replicates. The *X*-axis represents sampling days in the SE process: (**A**) *IPT1*; (**B**) *IPT5*; (**C**) *CKX3*; (**D**) *CKX9*; (**E**) *ARR2*; (**F**) *ARR9*. The relative transcript abundance was normalized using the *C. canephora* ubiquitin gene. Error bars represent standard error (*n* = 3). Different letters represent the statistical significance of mean differences between each determination at a given time according to the Tukey test (*p* < 0.05). For (**B**,**F**), there was no significant difference.

To validate the results obtained during the transcriptome analysis, we quantified the expression of the genes *IPT*, *CKX*, and *ARR* by real-time quantitative PCR (RT-qPCR). The expression of *CcIPT1* increased sharply during the first minutes of the induction of the SE and then back to the basal level shown through the pretreatment (Figure 3A). On the other hand, *CcIPT5* was slightly induced after the first 5 days of the pretreatment and remained at those levels until day 21 (Figure 3B). The expression of *CcCKX3* (Figure 3C) and *CcCKX9* (Figure 3D) genes presented a very similar pattern from the beginning of the pretreatment until two days after the induction of SE. They began their expression only after the induction of SE. Their expression increased sharply for the first 48 h and then decreased.

Phylogenetic analysis showed that *CcARR2* belongs to the ARR Type-B family and *CcARR9* belongs to the ARR Type-A family Figure 1). RT-qPCR analysis showed a slight increase in the expression of *CcARR2* during the first minutes after the induction of SE and then increased dramatically during the following 48 h. Its expression decreased by half by day 21 (Figure 3E). By contrast, the expression of the *CcARR9* gene was expressed constitutively throughout the SE process. The expression of this gene doubled after five days in the pretreatment, decreased slightly in the subsequent days, and then stayed at this level until minutes after the induction of SE. After that, the expression returns to the initial level (Figure 3F).

### 2.4. CK and Auxin Quantification

**Figure 4:**
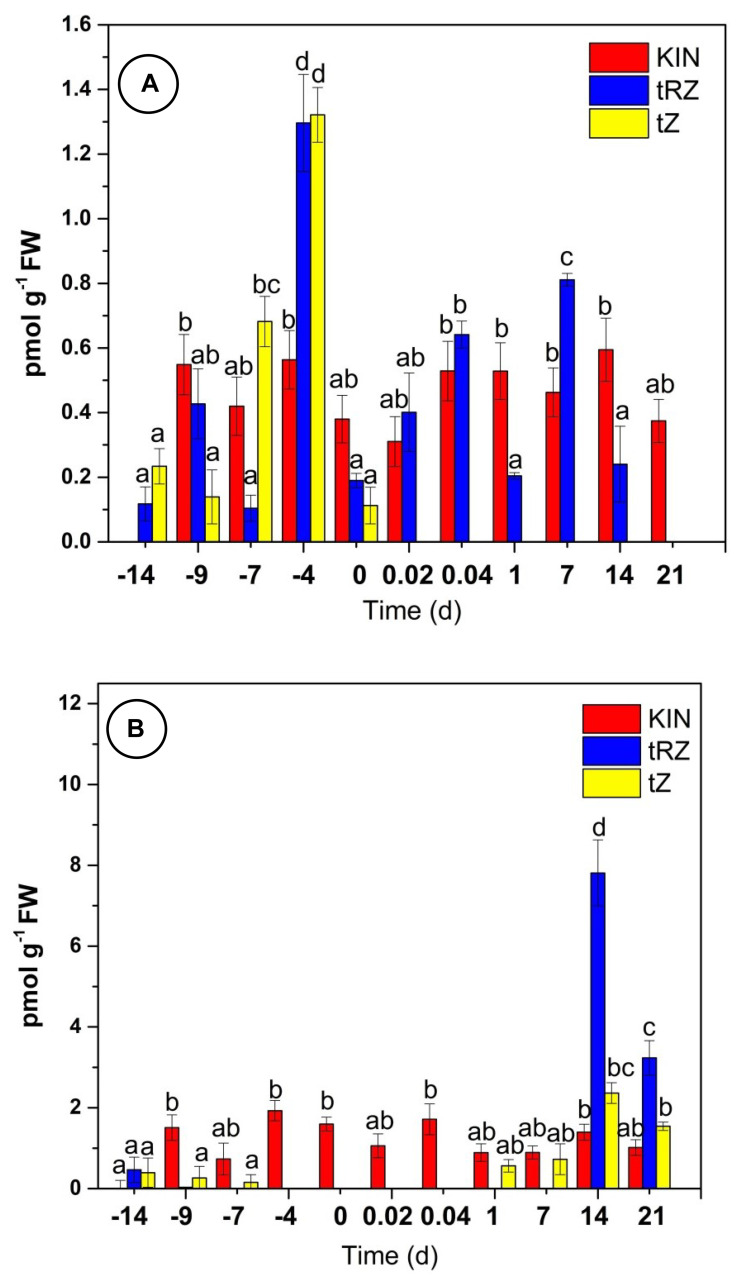
Endogenous CK content during SE in *C. canephora*: (**A**) without L-Kyn; (**B**) with L-Kyn. A total of 100 mg of tissue was collected from the beginning of the preconditioning of the plantlets (days -14, -9, and -4) to the induction day (day zero). Samples were collected after the induction (0.02, 0.04, 1, 7, 14, and 21 d) of SE. For the L-Kyn experiment, the inhibitor (1 µM) was added at the beginning of the pre-treatment. The chromatographic system is described in Materials and Methods. All analyses were performed with three biological replicates from two independent experiments. FW, fresh weight; bars display standard error (*n* = 3); KIN (red); tZ (yellow); and tZR (blue). Different letters represent the statistical significance of mean differences between each determination at a given time according to the Tukey test (*p* < 0.05).

Using three different standards, we confirmed the identity and quantified the CK endogenous content (tZ and tZR) and one analog (KIN) during the SE process. CK quantification presented very peculiar dynamics throughout SE of *C. canephora* (Figure 4A). The initial concentrations tZ (0.233 nmol g^−1^ FW) and tZR (0.117 nmol g^−1^ FW) reached their maximum concentration at -4 days (tZR, 2.596 nmol g^−1^ FW; tZ, 1.921 nmol g^−1^ FW) of pre-treatment and a considerable decrease on the day of induction (zero day). During the induction of SE, tZ was not detected; however, tZR (0.811 nmol g^−1^ FW) increased its concentration on post-induction days until day 7 and then decreased until it was not detectable by day 21.

In the case of kinetin (KIN), as expected, it was not detected at -14 d. However, it was present throughout the process of SE, reaching its highest concentration (0.963 nmol g^−1^ FW) on day -4 of the pre-conditioning and gradually decreasing in the early hours of the induction stage (Figure 4A).

### 2.5. Inhibiton of Aux Biosynthesis by L-Kynurenine

To further probe the cross talk between Aux and CK, we used an inhibitor of the biosynthetic pathway of Aux, L-kynurenine (L-Kyn), which inhibits the activity of the TAA1 enzyme, which is involved in the IPyA pathway in Aux biosynthesis [68]. We hypothesized that if Aux represses CK biosynthesis, decreasing Aux concentration will increase CK concentration. We used 1 µM L-Kyn only during the pre-treatment (Figure 4B).

We found tZR to be present at day -14. In the presence of L-Kyn, tZR decreased below detection levels from day -9 to day 14 after induction, when it showed a significant increase in its concentration, up to 8 pmol g^−1^ FW. Seven days later, it decreased to a little less than half. On the other hand, tZ was detected from -14 to -7 days of pre-treatment; no tZ was detected from -4 to 1 day. KIN was detected on day -9 and showed an increase (twice compared to the plants without L-Kyn treatment) up until the first hour after induction and a reduction in its content after that. The number of somatic embryos produced in control was 273 embryos per flask with 5 explants after 56 days, divided into 142 globulars, 93 heart-types, 30 torpedo-types, and 8 cotyledonary embryos. In the treatment with 1 µM of L-Kyn, there were no embryos, and only callus was formed (Figure S2), while with 0.1 µM, callus was formed and less than 10 globular embryos.

### 2.6. Inhibiton of CK Biosynthesis by Pravastatina

**Figure 5:**
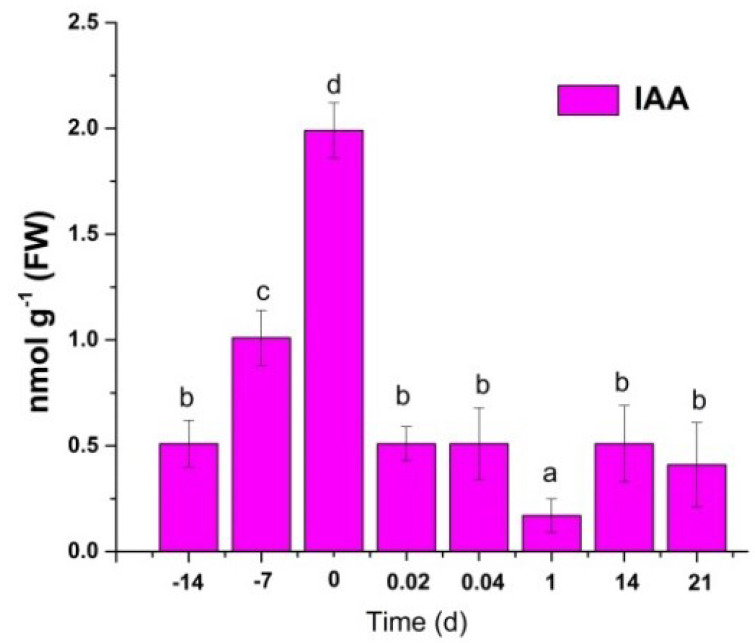
Endogenous auxin content during SE in *C. canephora*. A total of 100 mg of tissue was collected from the beginning of the preconditioning of the plantlets (days -14, -9, and -4) to the induction day (day zero). Samples were collected after the induction (0.02, 0.04, 1, 7, 14, and 21 d) of SE. The chromatographic system is described in Materials and Methods. All analyses were performed with three biological replicates from two independent experiments. FW, fresh weight; bars display standard error (*n* = 3). Different letters represent the statistical significance of mean differences between each determination at a given time according to the Tukey test (*p*< 0.05).

As expected, auxin content increased during pretreatment and decreased sharply during the first hours of SE induction (Figure 5).

**Figure 6:**
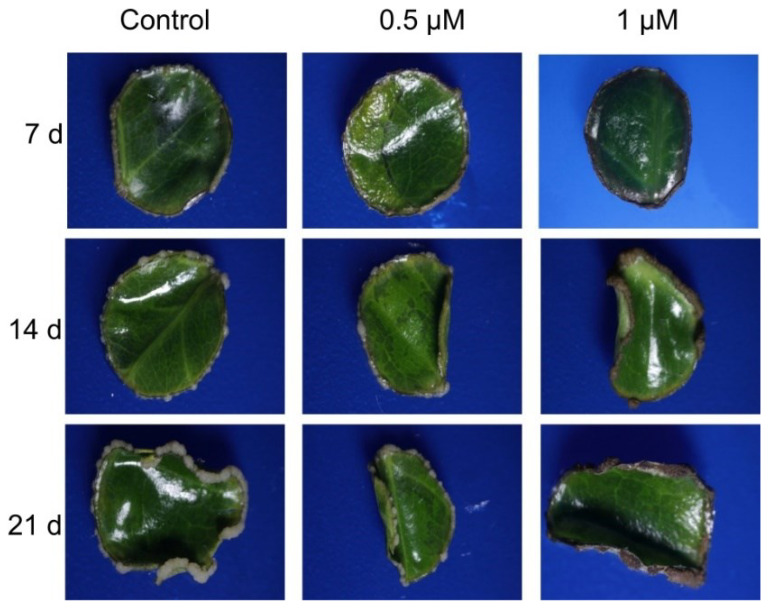
Induction of SE in *C. canephora* in the presence of pravastatin (PVS). The SE process was performed as described in the methodology with some modifications. PVS was added only in the preconditioning stage.

Pravastatin (PVS) was used to analyze the dynamics of the CK in CK-inhibiting conditions. PVS inhibits the activity of the enzyme HMGR (3-hydroxy-3-methylglutaryl-CoA reductase). PVS was employed during the pre-treatment at 0.5 and 1 µM. In Figure 6, we present the response of the explant to the PVS treatment. With both concentrations of PVS, the proembryogenic mass is less abundant than in the control explants, suggesting that PVS affects the SE in *C. canephora* and demonstrates the CK’s crucial role during the SE process.

**Figure 7:**
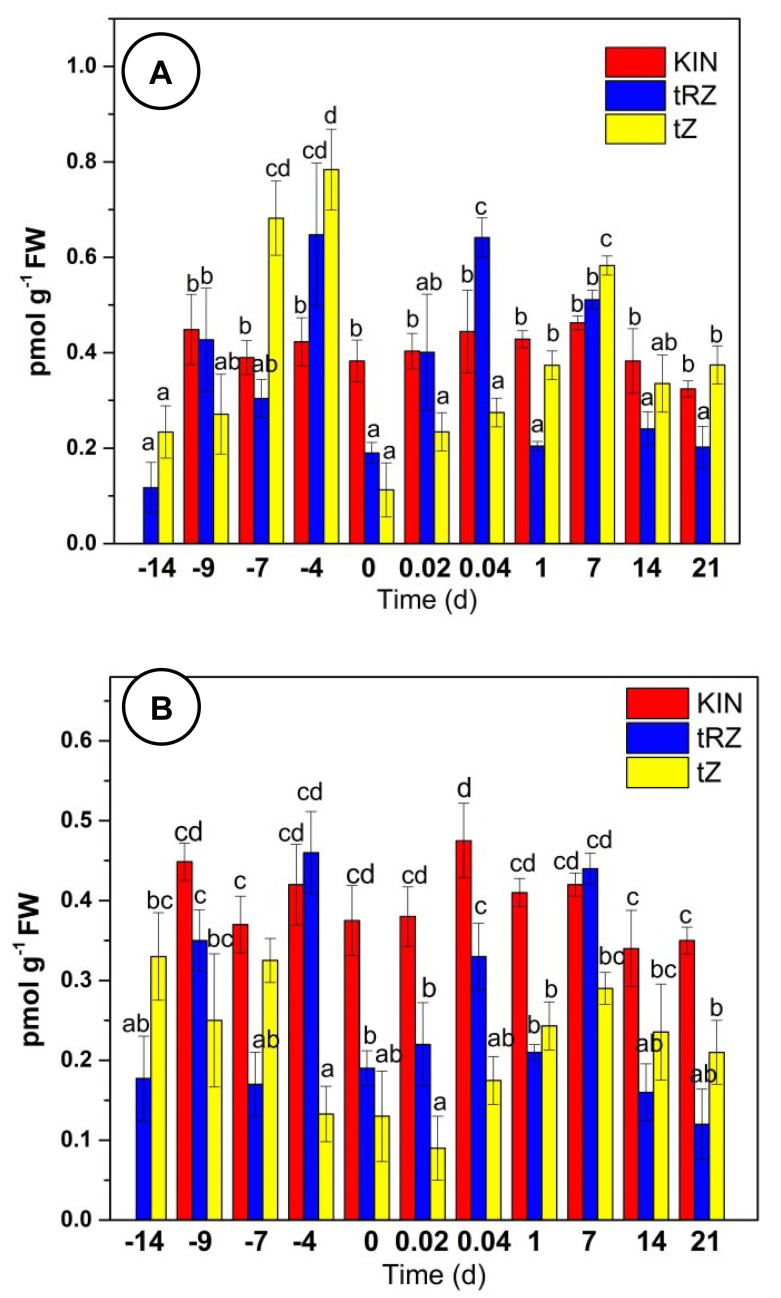
Endogenous CK content with CK inhibitor PVS. Preconditioning seedlings (-9, -7, -4, and 0 d) were supplemented with 0.5 µM (**A**); and 1 µM PVS (**B**). The chromatographic system is described in Materials and Methods. All the analyses were performed with three biological replicates. FW, fresh weight; error bars display standard error (*n* = 3); KIN (red); tZ (yellow); and tZR (blue). Different letters represent the statistical significance of mean differences between each determination at a given time according to the Tukey test (*p*< 0.05).

The effect of PVS on the dynamic of CK concentrations is shown in Figure 6. The presence of PVS decreases CK concentration substantially. After 10 days in the presence of the inhibitor, the amount of tZR and tZ decreased by 28.8% and 81%, respectively. The effect was maintained after SE induction. As expected, the concentration of KIN was unchanged in both treatments (Figure 7a,b).

**Figure 8:**
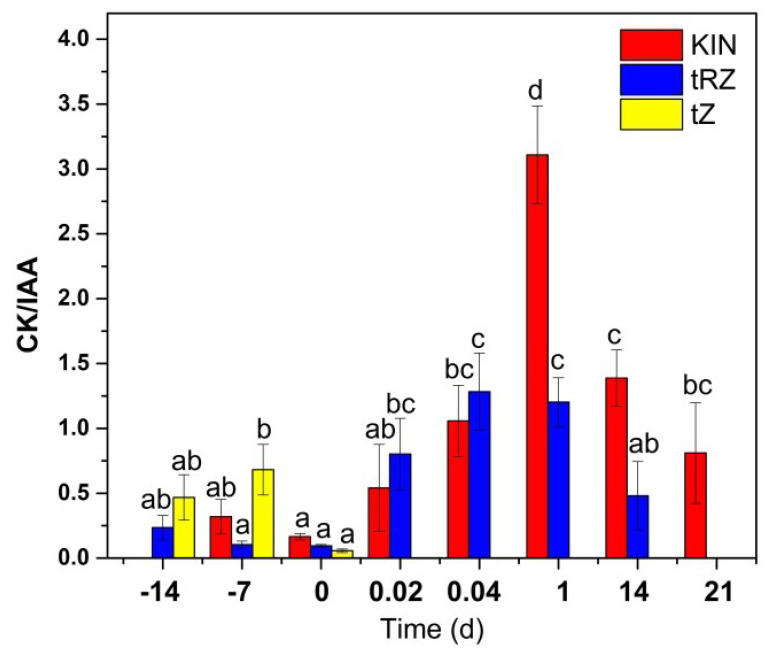
CK/IAA ratio during somatic embryogenesis of *C. canephora*. > 1, major CK/IAA ratio. < 1, minor CK/IAA. KIN/IAA (red), tZ/IAA (yellow), and tZR/IAA (blue). Different letters represent the statistical significance of mean differences between each determination at a given time according to the Tukey test (*p*< 0.05).

The dynamics shown by Aux and CK suggest that the relationship between them changes significantly throughout the process of SE induction. Using the data in Figure 4a, we calculate the relationship of each CK with the amount of IAA (Figure 8). In the case of CK, we consider the KIN, which, although it is not a natural CK, enters the explant from the culture medium. We have previously shown that without the presence of KIN in the pretreatment medium, the SE process essentially does not take place [69].

As expected, at the beginning of the pretreatment, there is no KIN. The ratio of tZ/IAA increases during the first week and then decreases at the time of induction since tZ practically disappears from the explant tissues. The ratio of tZR/IAA is low during pretreatment; however, the ratio increases during the first 24 h of induction, decreases at 14 days, and tZR is no longer detectable by day 21 after induction. In the case of the relationship KIN/IAA, it can be seen that after remaining low during pretreatment, a very significant increase is produced at the induction of SE. This increase peaks on day 1 after induction and decreases to a third of its peak by day 21 but is still significant (Figure 8).

## 3. Discussion

**Figure 9:**
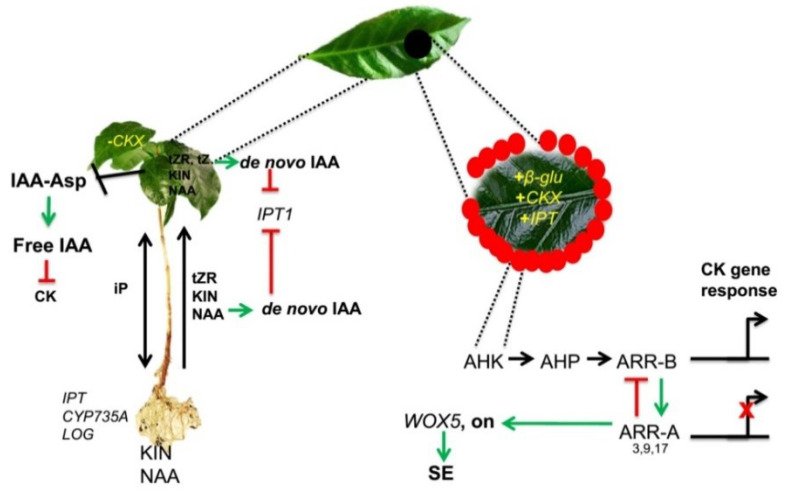
Proposed CK homeostasis model during somatic embryogenesis of *C. canephora*. NAA and KIN are transported into the leaves. KIN probably are acropetally transported by an ENT transporter using the xylem. NAA can induce de novo Aux biosynthesis; tZR and tZ are synthesized in the roots and transported to the aerial part, while iP is synthesized in the roots and aerial part. The tZ can induce de novo Aux biosynthesis as well. This leads to IPT1 repression. NAA probably represses *CKX* genes, increasing the CK content in the plantlets. It has been demonstrated that CK can be compartmentalized in vacuoles in their conjugated form. BA (red circles) induces the expression of *CKX* and *IPT* genes. BA induces *β-GLUCOSIDASE* genes; hence β-glucosidase breaks the glycosidic bond and releases the free CK. The degradation products of KIN can be used to synthesize other CK. BA may activate the phosphorelay signaling system, leading to transcription of CK-related genes by *ARR* Type B or repression of CK-related genes by *ARR* Type A. *ARR* Type A can downregulate Aux repressing *PIN1* genes through SHY2/IAA3 induction. However, CK can induce de novo synthesis, inhibiting the IAA conjugation with aspartic amino acid.

The data from this research suggests a model for CK’s role during SE in *C. canephora* (Figure 9). First, the preconditioning medium contains two PGRs, NAA 0.54 µM, and KIN 2.32 µM. These regulators are absorbed through the vascular system’s roots and transported to the leaves. In the case of CK, our transcriptome data indicates the expression of two genes codifying for putative types of transporters (*CcPUP* and *CcENT*).

CK quantification indicates that tZ and tZR increase their concentration days before the induction stage (Figure 7A). This increase may be due to the presence of KIN, which is used in the pretreatment medium, as it has been suggested that CK induce Aux biosynthesis in *A. thaliana* [70] and regulate local Aux metabolism [14,15,71].

Meanwhile, NAA quickly enters the cell by diffusion but needs auxin efflux transporters, probably PIN8, to reach the apoplast [72,73]. KIN is absorbed and compartmentalized [74], perhaps in a storage form. It has been demonstrated that adenine from the degradation of KIN can be recycled and used for the formation of other cytokinins [75].

The RT-qPCR dates showed that *CcIPT* genes are being repressed, perhaps by IAA produced de novo by NAA (or by NAA *per se*), which probably affects tZ and tZR. The action of NAA suppresses the *CcCKX* genes. Signaling and perception are probably being affected at this point; NAA and KIN induce the *ARR* Type-B genes, and NAA represses *ARR* Type-A genes. Therefore, the CK-related genes are transcribed. Among these is the *CcIPT5* gene. It has been demonstrated that CK induce *ARR1* (Type B) and, consequently, *SHY2/IAA3*, a repressor of *PIN*. Thus, CK can modulate the transport of *PIN* [19,76].

AHKs-ARR1/12 mediated Aux biosynthesis by up-regulating *TAA1* and *YUC8* by up-regulating ASB1 [77]. This regulation occurs through PIF4, which CK also induces through AHKs-ARR1/12 [78]. To understand the interaction between Aux and CK, it is essential to consider that most of these genes are families. An example of the complexity that this fact introduces is that in the apex of the adventitious root of *A. thaliana*, CK mediate the up-regulation of *YUC6*, which is involved in the formation of the quiescent center [79]. While in gynoecia primordium, CK induce the expression of *YUC1* and *YUC4* to ensure correct domain patterning [31]. What further shows the complexity of the system is that CK also modulate Aux degradation. *ARR1* binds directly to *GH3.17* and activates its transcription in response to the presence of CK, thus promoting the sequestration of free Aux to the conjugated form [80]. In addition, IAA [81] increases when explants are exposed to NAA. In the case of *C. canephora*, Uc-Chuc et al. [81] showed that the increase is due to de novo synthesis.

In the induction phase, explants are placed in a medium with 5 µM BA. Transcriptome data demonstrate an increased expression of the *β-GLU* gene family in the induction (Figure 3). It has been shown that CK in Arabidopsis presents 100% in conjugated form [82]. The β-glucosidase (β-Glu; EC 3.2.1.21) cleaves the β-glucosidic linkages of *O*-glucosides of CK into the active form [49,50]. The β-glucosidases families are very abundant in plants. There are 47 genes in *A. thaliana*, 38 in *O. sativa*, and 47 in the genome of *C. canephora*. Our results suggest that the cell requires a more significant amount of CK and succeeds in breaking the glycosidic bond from conjugates of CK, leaving the CK in its free form (active form).

Transcriptome data and RT-qPCR of *ARR* genes during the induction were very similar; *ARR9* showed increased expression in the first days after induction, and *ARR2* remained constitutively expressed; throughout the SE process its expression coincided with the increase in the expression of ARR. Data from RT-qPCR indicate an increase in the expression of *CcCKX* genes in the first days after induction (Figure 3). Meanwhile, the *IPT* genes were expressed constitutively (*CcIPT5*) or presented an increase in the first hours (*CcIPT1*) (Figure 3).

Our results suggest that proteins of the CcIPT family share a higher degree of amino acid sequence homology with other plant species, indicating a greater phylogenetic closeness (Figure 1). Moreover, our data propose that the expression of *IPT* and *CKX* are controlled by the *ARR*-type genes and the upstream signal of these genes. The data from the transcriptome indicate a strong expression of *AHK* and its homolog *CKI1* [67] during the induction.

Vyroubalová et al. [83] demonstrated that *ZmIPT* and *ZmCKX* genes (in leaf explants) can be induced (*IPT1-5-8*; *CKX1-2-4-8-10-11*) or repressed (*IPT10*; *CKX3-6-9-12*) in a medium with 10 µM BA. These results suggest the differential response of *IPT* and *CKX* genes to BA. It has been observed that Aux controls the CK biosynthesis in Arabidopsis (in roots) by activation of *IPT5* and *IPT7* genes [33], while in pea, the expression of two CK biosynthesis genes (*PsIPT1* and *PsIPT2*) are negatively regulated by Aux [84].

Similarly, Liu et al. [85] quantified the *IPT* and *CKX* gene expression in Brassica and showed that five *BrIPT* genes are induced with BA (100 µM), and three *BrIPT* genes are repressed 30 min and 1 h after treatment, respectively. On the other hand, the same author showed that 11 *BrCKX* genes respond positively to BA (100 µM), and 3 *BrCKX* genes are repressed. Brugière et al. [86] inferred that the CKX gene can be induced by BA but is clearly affected by 2, 4-D. Werner et al. [87] showed that CKX gene increases its expression with BA but is repressed with 1-naphthaleneacetic acid (NAA)

In this research, we suggest that NAA has a negative effect on the *CcCKX* gene, but BA has a positive effect on the expression of the *CcCKX3* gene. In our work, *CcIPT1* gene was repressed during the pre-treatment, perhaps by the presence of NAA. Others have demonstrated the de novo synthesis of IAA by NAA in C. canephora [45]; it is likely that the synthesis of de novo IAA represses the expression of the *CcIPT1* gene. The *CcIPT5* gene was expressed constitutively during the whole SE process. Thus, we suggest that the *CcIPT5* gene is probably crucial for CK biosynthesis during SE in *C. canephora*.

It has been shown that *ARR* Type-A (3 and 4) genes induce its expression in the presence of zeatin or BA. Nevertheless, *ARR* Type-B genes (1, 2, and 10) are induced in the same way with/without zeatin or BA [88], suggesting that *ARR* Type-B genes can be constitutively expressed. The same authors demonstrate the expression of the *ARR3* gene (Type A increases) and the *ARR10* gene (Type B decreases) in a medium with 2, 4-D 100 µM. These results suggest that analogs of Aux can play an important role in *ARR* gene expression. Argyros et al. [89] showed that ARR Type A is induced by BA and simultaneously showed that the *ARR* Type-A gene depends on *ARR* Type-B genes for its expression.

The *AHP* genes also increase their expression in the same step. It is known that CK act as a signal molecule that activates the phosphorelay system in the cascade of CK perception/signal [11]. This signal is recognized by the *AHK*, which are autophosphorylated and transfer the phosphoryl group of the AHP proteins. The transcriptome and RT-qPCR show that *ARR* Type A represses *ARR* Type B. These proteins function as mobile elements in CK signaling to transfer the phosphoryl group signal from the endoplasmic reticulum to the nucleus, responsible for phosphorylating *ARR* Type-B genes, which promote transcription of genes related to CK. We assume that BA is taken in tiny amounts, maybe as femtomolar, as in other species [90]; however, that amount is sufficient to confer the signal to the phosphorelay system. This finding may suggest that the crucial step is upstream of the *ARR* genes, as *AHK* and *AHP* are highly expressed in the early induction days. These provide signals for the transcription of *ARR* Type-B genes.

The first studies on the mobility of this CK analog show that kinetin riboside (KR) exceeds the mobility of KIN and BA in xylem [91]. It has been shown that KIN can be quickly absorbed by the plant [92] and can be compartmentalized. When it is degraded, the AMP resulting from the removal of the furfuryl side-chain type may be used for the formation of endogenous CK [74,75]. In this work, we hypothesize that KIN is transported from the roots to the leaves by a system of long-distance transport, probably by CcENTN3; KIN is then rapidly absorbed into the leaves and compartmentalized, and when leaf explants are removed from the seedling, the stored KIN is released gradually due perhaps to the absence of tZ in the induction stage and may function as a signal molecule. We suggest that L-Kyn disrupts the balance of endogenous CK, and the plant cells need a single stimulus to trigger the SE. This breaks up endogenous CK, forcing the explant to take KIN and metabolize it (Figure 4).

In our system, the globular structures appear at 21 days [69], coinciding with the increase and subsequent decrease in the CK/IAA ratio (Figure 7). Wang [93] reports an increase in the CK content is necessary to induce SE in Arabidopsis, but CK per se activates ARR7 and ARR15 (CK repressors) to decrease CK and induce WOX5 for the RAM establishment.

In summary, in our model, we propose that NAA and KIN are transported into the leaves. KIN probably are acropetally transported by an ENT transporter using the xylem. NAA can induce de novo Aux biosynthesis. Both tZR and tZ are synthesized in the roots and transported to the aerial part, while iP is synthesized in the roots and aerial part; tZ can induce de novo Aux biosynthesis as well. This leads to *IPT1* repression. NAA probably represses *CKX* genes, increasing the CK content in the plantlets. It has been demonstrated that CK can be compartmentalized in vacuoles in their conjugated form. BA induces the expression of *CKX* and *IPT* genes. BA induces *β-GLUCOSIDASE* genes; hence β-glucosidase breaks the glycosidic bond and releases the free CK. The degradation products of KIN can be used to synthesize other CK. BA may activate the phosphorelay signaling system, leading to transcription of CK-related genes by *ARR* Type B or repression of CK-related genes by *ARR* Type A. *ARR* Type A can downregulate Aux, repressing *PIN1* genes through SHY2/IAA3 induction. However, CK can induce de novo synthesis, inhibiting the IAA conjugation with aspartic amino acid.

## 4. Materials and Methods

### 4.1. Biological Material and Somatic Embryogenesis Induction

The SE induction process was carried out from plants of *C. canephora* Pierre var. Robusta cultured in vitro. [69]. Somatic embryos were germinated in maintenance medium composed by Murashige-Skoog [94] salts (MS: PhytoTechnology Laboratories, M524) supplemented with 29.6 µM thiamine-HCl (Sigma, C-8277; St. Louis, MO, USA), 550 µM myo-inositol (Sigma, I5125; St. Louis, MO, USA), 0.15 µM cysteine (Sigma, C8277; St. Louis, MO, USA), 16.24 µM nicotinic acid (Sigma, N4126; St. Louis, MO, USA), 9.72 µM pyridoxine-HCl (Sigma, P9755; St. Louis, MO, USA), 87.64 mM sucrose (Sigma, S539; St. Louis, MO, USA) and 0.285% (*w/v*) Gellan gum (PhytoTechnology Laboratories, G434, Lenexa, KS, USA). All culture media pH was adjusted to 5.8 and sterilized for 20 min, at 121 °C and 1 kg cm^−2^. After germination, the seedlings were grown under photoperiod conditions of 16 h light/8 h dark (150 µmol m^−2^ s^−1^) at 25 ± 2 °C. When the plantlets had six pairs of leaves, they were preconditioned for 14 days in a semi-solid MS [94] medium supplemented with 2.32 µM kinetin (Sigma, K0753; St. Louis, MO, USA) and 0.54 µM NAA (Sigma, N-1145; St. Louis, MO, USA) under the same conditions as described above. Samples were collected for further analysis at −14, −9, and 0 days before the induction of SE and 0.02, 0.04, 1, 2, and 21 days after SE induction. After the 14 days in the preconditioning stage and under aseptic conditions, circular explants were cut with an eight mm diameter punch, using the second and third pair of leaves of plants as starting material since the first two pair of leaves have shown poor embryogenic response [69]. Five circular explants were incubated in 250 mL flasks containing 50 mL of induction liquid medium. Induction medium was composed by Yasuda [95] salts with the modified nitrogen source, and 5 µM of BA added (PhytoTechnology Laboratories, B800, Lenexa, KS, USA). All flasks were incubated at 25 ± 2 °C and 55 rpm for 56 days protected from light.

After 14 days in the pre-treatment medium, the explants are placed in the induction medium supplemented with benzyladenine (BA, 5 µM); 21 days after the start of induction, the explants begin to show small globular structures. At 28 and 35 days, the globular structures develop into the heart- and torpedo-type embryos; 42 days after induction, the first cotyledonary embryos appear. At 56 days after induction of somatic embryogenesis, a mixture of all four developmental stages can be observed.

The documentation of SE induction and the development of the somatic embryos were carried out using a stereoscopic zoom microscope (Nikon, SMZ745T, Tokyo, Japan) equipped with a photographic camera (Canon EO5 Rebel T3i, Tokyo, Japan).

### 4.2. Treatments with L-Kynurenine

L-kynurenine (L-Kyn), an inhibitor of the TAA1, the first enzyme involved in a two-step pathway to IAA biosynthesis, was added to the induction medium at a concentration of 1 µM, and CK concentration was determined during the pretreatment at −14, −9, −7, −4 and 0 d.

### 4.3. Analysis of the IPT, CKX, and ARR Genes

The amino acid sequences of IPT, CKX, and ARR derived from *Arabidopsis thaliana*, *Brassica napus*, *Capsicum annuum*, *Citrus sinensis*, *Daucus carota*, *Fragaria vesca*, *Ipomoea triloba*, *Jatropha curcas*, *Nicotiana attenuata*, *Oryza sativa* Japonica, *Phaseolus vulgaris*, *Pisum sativum*, *Prunus dulcis*, *Solanum lycopersicum*, *Theobroma cacao*, and *Zea mays*, together with the CcIPT, CcCKX, and CcARR proteins, were used for phylogenetic analysis. These se-quences were consulted from the National Center for Biotechnology Information Gen-bank Database, except for *C. canephora* sequences downloaded from Coffee Genome Hub [96]. The accession number of each consulted plant species is shown in Figure 1. Amino acid sequences were aligned using the MUSCLE algorithm with default parameters. The phylogenetic trees were performed with MEGA 7.0 software [97] using the neighbor-joining method (1000 bootstrap replicates).

### 4.4. Transcriptome Analysis

A manual search of the genes involved in the metabolism of CK was carried out by keyword within the transcriptome previously prepared in our laboratory, corresponding to the SE induction process [46]. Genes differentially expressed at different points in the SE process were identified, taking day −14 as a control. The heatmap was created using R software.

### 4.5. Design of the Primers

Specific primers were designed for each gene of interest following the *C. canephora* genome [98]. Primers for RT-qPCR were designed using the Primer 3 Plus tool [99]. On the other hand, some parameters were established such as the length (20–30 bp), GC content (45–60%), melting temperature (59–60 °C), and the amplicon size of the primers (150 and 200 bp). Other parameters such as formation of secondary structures and dimerization were verified with the OligoAnalyzerTM online tool [100]). Primers information is listed in Table S2.

### 4.6. RNA Extraction

For this study, total RNA was obtained from 100 mg of leaf tissue (approximately seven to nine explants) and the Direct-zol RNA MiniPrep Extraction Kit (Zymo research, R2051, Irvine, CA, USA). All steps were carried out at low temperatures to avoid RNA degradation. RNA quality was determined and quantified using a NanoDropTM 2000 spectrophotometer (Thermo Scientific, Waltham, MA, USA), and the integrity was observed by 1.5% agarose gel electrophoresis. From 500 ng of total RNA, complementary DNA synthesis was carried out using the SuperScripTM First-Strand Synthesis Kit (Invitrogen, 11904018, Frederick, MD, USA). The cDNA templates for qPCR amplification were prepared from 3 individual samples from 3 independent experiments for each condition.

### 4.7. Real-Time Quantitative PCR (RT-qPCR Analysis)

An Eco Real-Time PCR System (Illumina, EC-900-1001, El Monte, CA, USA) was used to perform the analysis with the following parameters: one initial cycle of 95 °C for 5 min, 40 cycles of denaturation at 95 °C for 15 s, and alignment and extension at 60 °C for 45 s. Each reaction contained 150 ng of cDNA template, 10 µM of each primer, and 1× EXPRESS SYBR™ GreenERT™ qPCR SuperMix (Invitrogen, 11784200, Frederick, MD, USA) in a final volume of 10 µL. The analysis of the results was performed with the Illumina EcoStudy V5.0 software. The expression data obtained were normalized with the ubiquitin reference gene, and the relative expression levels were quantified applying the 2^−ΔΔCT^ method [101].

### 4.8. Auxin and Cytokinin Extraction

For Aux and CK extraction, 100 mg of leaf tissue was obtained during the preconditioning stage (−14, −9 and 0 days) and after SE induction (0.02, 0.04, 1, 7, 14 and 21 days). The selected samples were frozen and stored at −80 °C until their use. The leaf tissue was pulverized with liquid nitrogen in a mortar until a fine powder was obtained, to which 1 mL of water acidified with HCl (pH 2.8) was added. The mixture was placed in a glass tube and 1 mL of acidified water, 1 mL of butylated hydroxytoluene (Acros Organics, 112992500, Thermo Fisher Scientific, Waltham, MA, USA) and 1 mL of ethyl acetate were added. The mixture was vigorously stirred for 1 min between the additions of each reagent. Then, 5 mL of ethyl acetate was added and agitated for another min, and centrifuged at 2000 rpm (Hettich ZENTRIFUGEN, Mikro 22 R, Buford, GA, USA) for 5 min. Three mL of the organic phase was taken and evaporated with nitrogen gas. The dry sample was suspended in 1 mL of the HPLC mobile phase system for CK (30% acetonitrile; J. T. Baker, 9017–03:70% water containing 0.5% acetic acid; CTR Scientific, 00500) and filtered through a Millipore filter (0.22 μM), or 1 mL of the HPLC mobile phase system for Aux (60% acetonitrile: 40% water acidified with 0.5% acetic acid), and filtered through a Millipore filter (0.22 µM). Analyzes were performed in triplicate of two independent experiments.

### 4.9. High-Performance Liquid Chromatography (HPLC)

For the analysis of the samples, an Agilent Technologies 1200 high-resolution liquid chromatography (HPLC) system consisting of a quaternary array of pumps (Agilent Technologies G1311A, Santa Clara, CA, USA) connected to an automatic injector (Agilent Technologies (G1329A) was used. A total of 20 µL of the tissue extract was injected and subjected to chromatography with an isocratic elution system with a flow rate of 1 mL min^−1^ in a C_18_ reverse-phase column (Phenomenex, Torrance, CA, USA) of 250 mm × 4.6 mm. The Aux were detected with a fluorescent detector (Agilent Technologies G1321A, Santa Clara, CA, USA) at an emission length of 280 nm and an excitation length of 340 nm. The CK were detected with a diode array detector (Agilent Technologies G1315B, Santa Clara, CA, USA) at an emission length of 280 nm.

### 4.10. Liquid Chromatography/Mass Spectrometry for Auxin and Cytokinin Identification

For identifying Aux and CK, we used a LC-MS/MS system which consists in a Thermo LTQ Orbitrap mass spectrometer equipped with heated-electrospray ionization source (HESI-II). Parameters in the positive ion mode were: sheath gas, 60; auxiliary gas, 20; source temperature, 310 °C; spray voltage, 4 kV. The chemical fragmentation of Aux and CK was determined from a solution of individual analytes concentrated to 100 µg mL^−1^ in methanol:water (80:20; *v/v*), which was directly infused at a flow rate of 5 µL min^−1^. The Collision energy dissociation (CID) condition was optimized to yield nearly 20% of the parent ion. LC separations were performed on a reverse-phase ZORBAX Eclipse XDB C_18_) column (150 × 4.6 mm i.d., 5 µm particle size, 80 Å pore size; Agilent Technologies, Wilmington, DE, USA) using a gradient of solvent A (0.1% formic acid in LC-MS water) and solvent B (0.1% formic acid in acetonitrile). The flow rate was set to 0.3 mL min^−1^, and 2 µL was injected. The gradient program for CK was set as follows: 5% solvent B, 0 min; 20% solvent B, 20 min; 30%, 32 min; 80%, 34 min; 100%, 36 min; 100%, 2 min; 5%, 40 min; 5%; 6 min. On the other hand, the gradient program for Aux was set as follows: 5% solvent B, 0 min; 40% solvent B, 27 min; 100%, 32 min; 100%, 2 min; 5%, 36 min; 5%, 6 min. Raw data obtained from the LC-MS system were processed with Xcalibur software (Thermo Scientific; Waltham, MA, USA; V 4.1).

### 4.11. Calibration Curves

Different calibration curves were made for the standards kinetin (KIN; Sigma 852 643; St. Louis, MO, USA), trans-zeatin (tZ; Sigma Z0876; St. Louis, MO, USA), and trans-zeatin riboside (tZR, SIGMA Z0375; St. Louis, MO, USA) to quantify the Aux and CK present in the tissues. The ranges for the calibration curves for tZ and kin ranged from 1–6 pmol; for tZR, they ranged from 0.5–4 pmol.

### 4.12. Statistical Analysis

The data processing to make the graphs and the statistical analyses were performed with the ANOVA variance analysis program using the Origin Pro 2017 64-bit software, ver. 94E (Data Analysis and Graphing Software). Significance values were determined using the Tukey test. The dierences were considered significant at *p* < 0.05.

## 5. Conclusions

The data presented in this work suggests that Aux and CK are fundamental PGRs to the proper development of SE of *C. canephora*. Previous investigation by our lab demonstrated the importance of Aux in the SE process. With the help of pharmacology and inhibiting crucial biosynthesis pathways, we demonstrated the individual importance of each PGR. Transcriptomic analysis and RT-qPCR helped us to understand genes’ expression in our SE process. Quantification results showed the complex dynamics of CK, and the identification validated our results.

## Data Availability

Not applicable.

## Supplementary Materials

The following are available online at https://www.mdpi.com/article/10.3390/plants11152013/s1, Figure S1: Expression profiles of selected CK biosynthesis and signal gene members in *Coffea canephora*. Figure S2: Induction of SE in *C. canephora* in the presence of L-Kyn. Figure S3: Chromatograms and fragmentation pattern obtained for trans-Zeatin (tZ) by LC-MS/MS., Figure S4: Chromatograms and fragmentation pattern obtained for trans-Zeatin Riboside (tZR) by LC-MS/MS, Figure S5: Chromatograms and fragmentation pattern obtained for Kinetin (K) by LC-MS/MS, Figure S6: Chromatograms and fragmentation pattern obtained for Indole-3-Acetic Acid (IAA) by LC-MS/MS, Table S1: Gene-specific primer sequences used for Quantitative Real-time RT-qPCR amplification, Table S2. Overview of tandem mass spectral and UHPLC features for the PGRs detected.
