# Supplementary material for: Auxin-Cytokinin Cross Talk in Somatic Embryogenesis of Coffea canephora
Source: Plants (Basel). 2022 Aug 2;11(15):2013. doi: 10.3390/plants11152013 (PMC9370429; doi:10.3390/plants11152013)
Supplement: Supplementary file 1 [file plants-11-02013-s001.zip › plants-1742743_supplementary.pdf]

plants

Supplementary Material

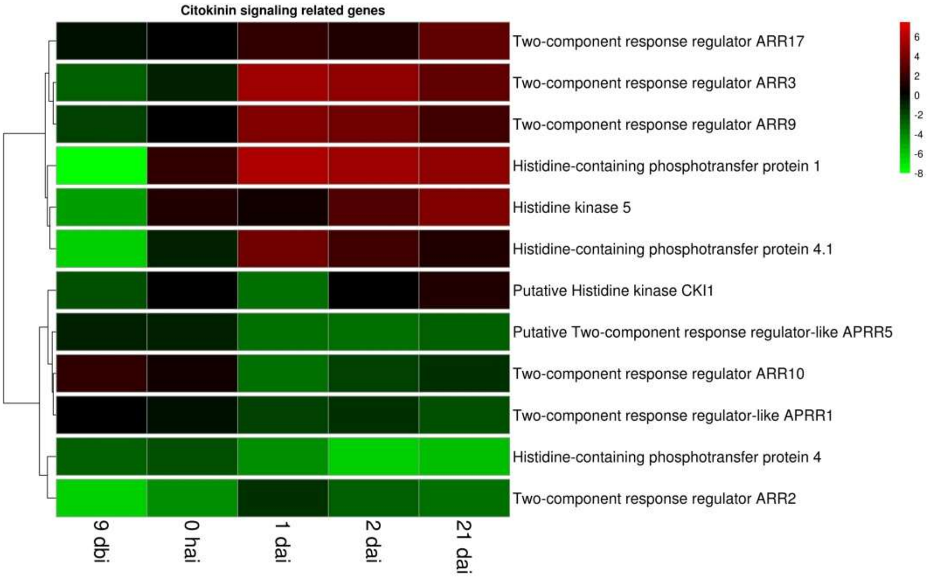

Figure S1. Expression profiles of genes involved in auxin and CKs signaling. The heatmap shows changes in transcript levels in different sampling points (-14 days vs. -9, 0, 1, 2, and 21 days). Gene names were annotated using Ugene and Blast2GO software. Change in expression during SE of C. canephora. Hierarchical clus-tering was used to group genes with similar expression profiles. The red color represents upregulated genes, and the green represents downregulated genes (log2 fold-change values).

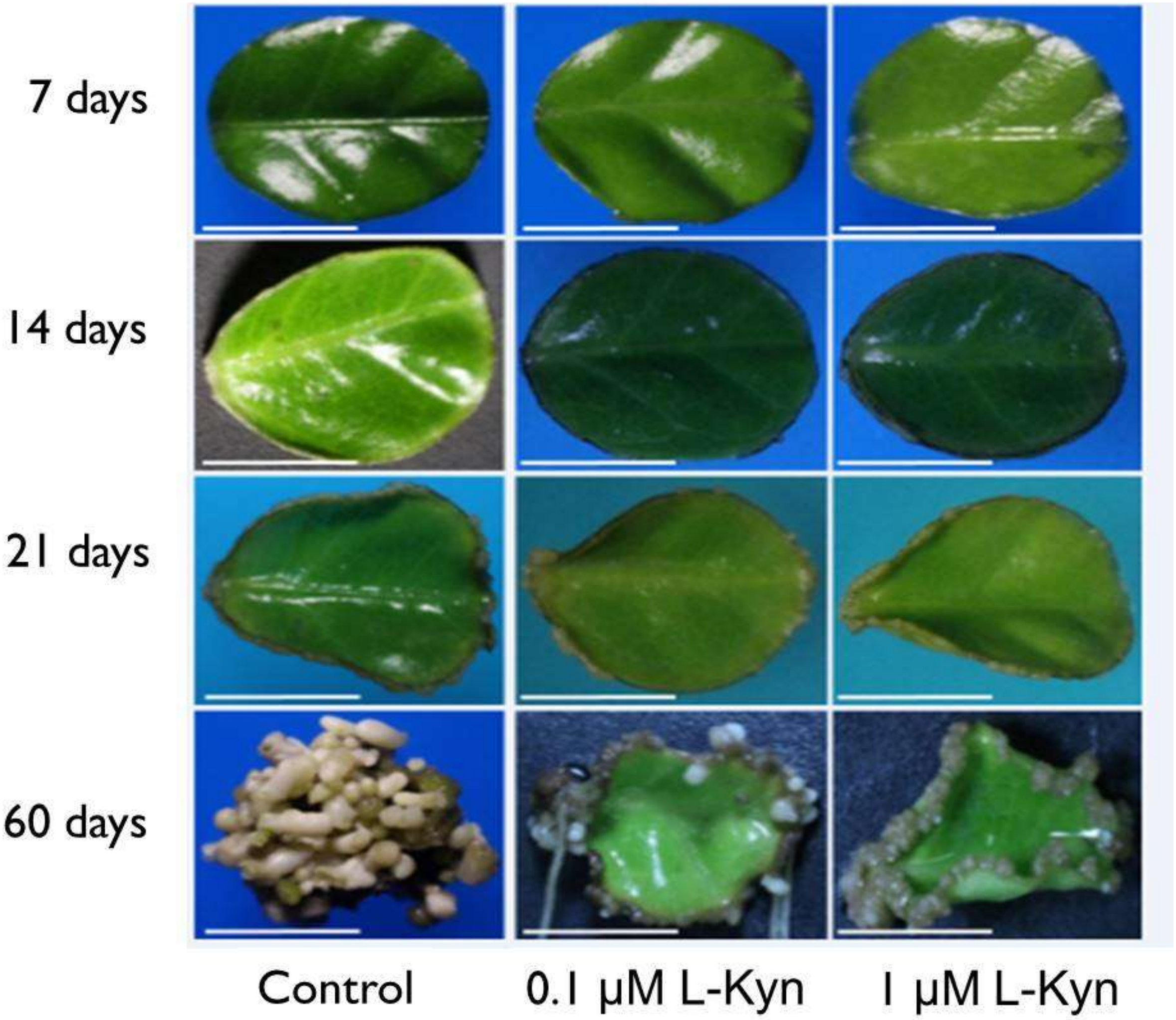

Figure S2. Induction of SE in *C. canephora* in the presence of L-Kyn. The SE process was performed as described in the methodology with some modifications. L-Kyn was added only in the preconditioning stage.

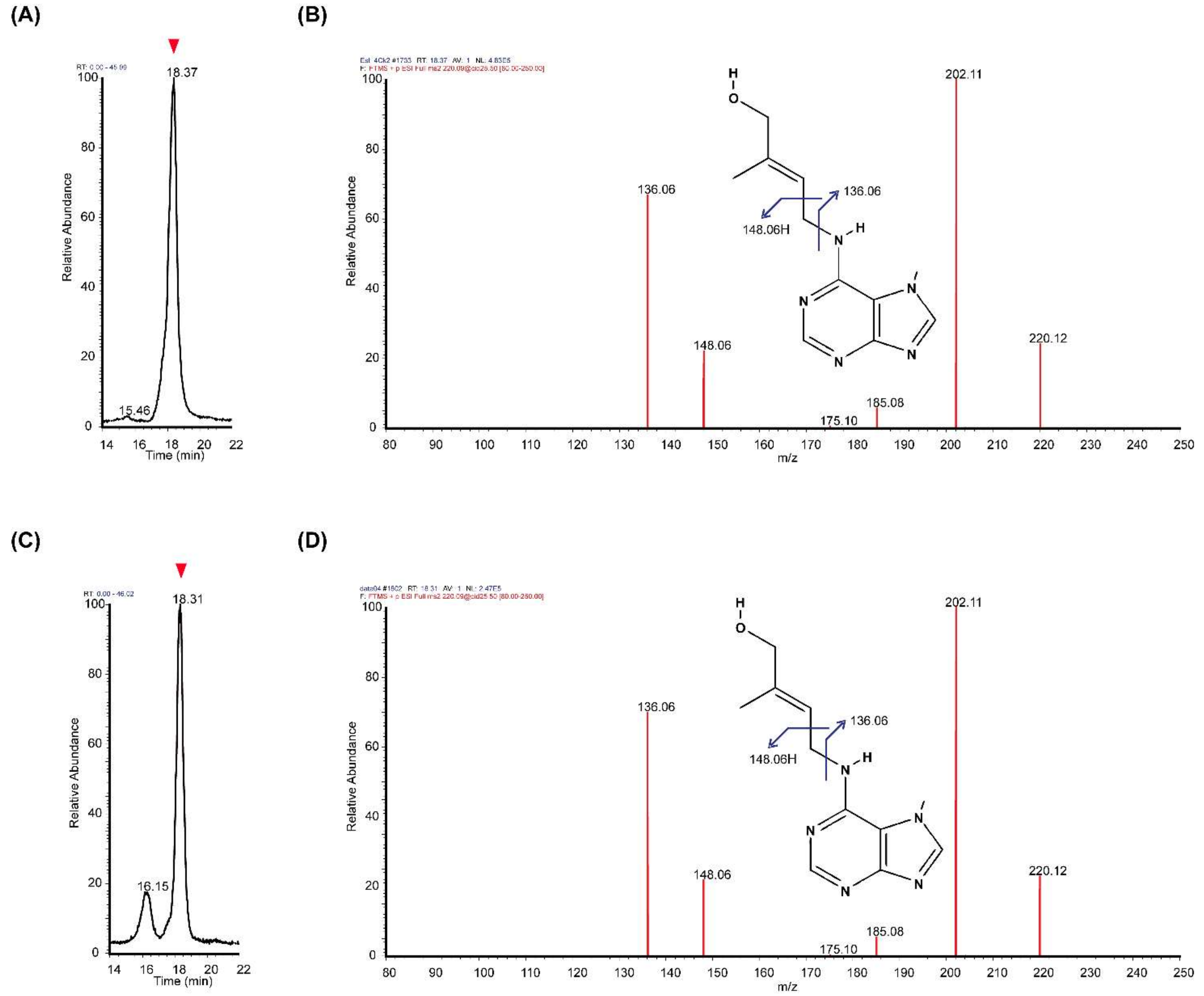

Figure S3. Chromatograms and fragmentation pattern obtained for *trans*-Zeatin (*t*Z) by LC-MS/MS. (**A** and **B**) Chromatograms and fragmentation pattern for analyte. (**C** and **D**) Chromatograms and fragmentation pattern obtained from pre-inducted leaf samples. Red arrowheads in chromatograms indicate the retention time for *t*Z.

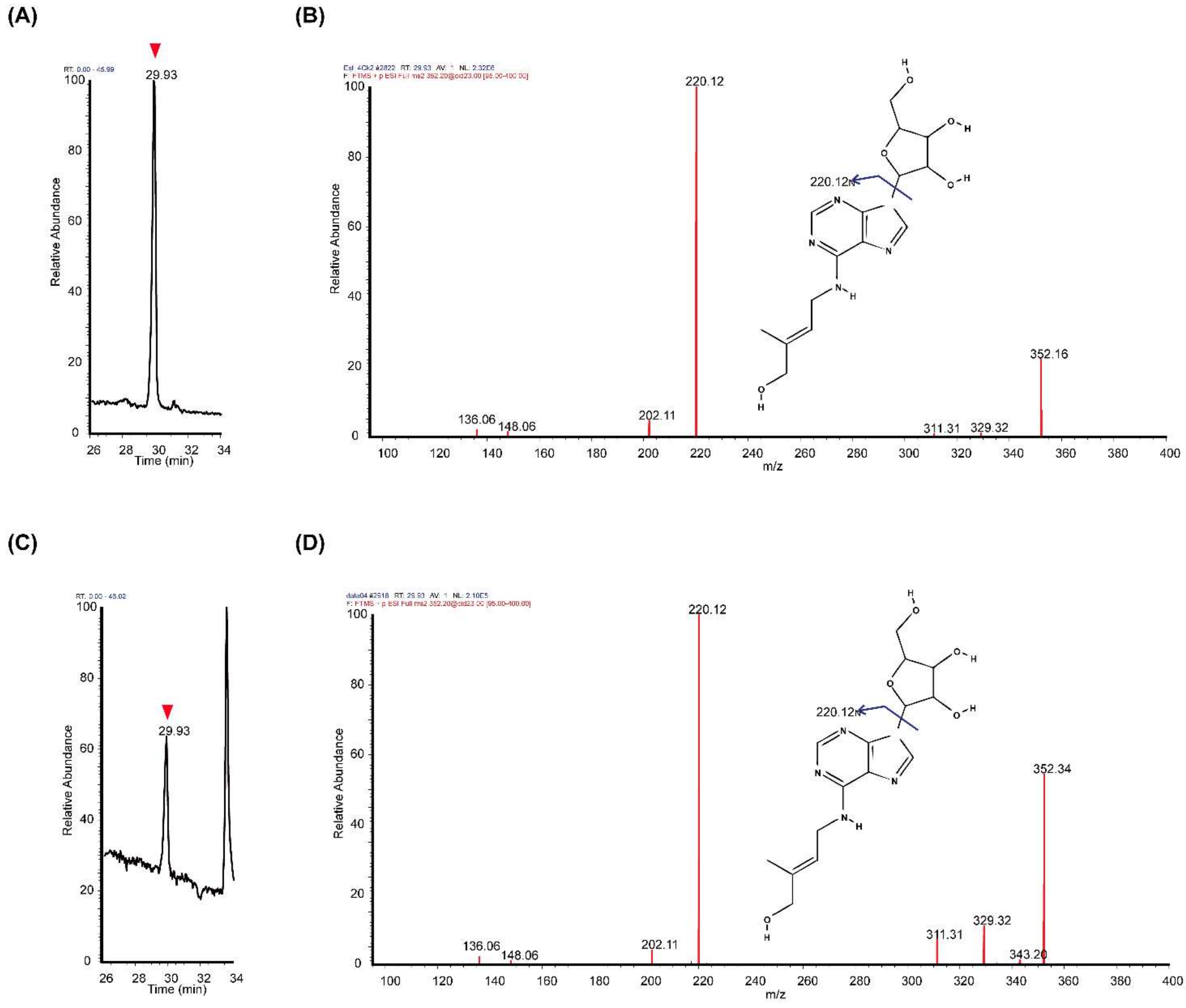

Figure S4. Chromatograms and fragmentation pattern obtained for *trans*-Zeatin Riboside (*t*ZR) by LC-MS/MS. (**A** and **B**) Chromatograms and fragmentation pattern for analyte. (**C** and **D**) Chromatograms and fragmentation pattern obtained from pre-inducted leaf samples. Red arrowheads in chromatograms indicate the retention time for *t*ZR.

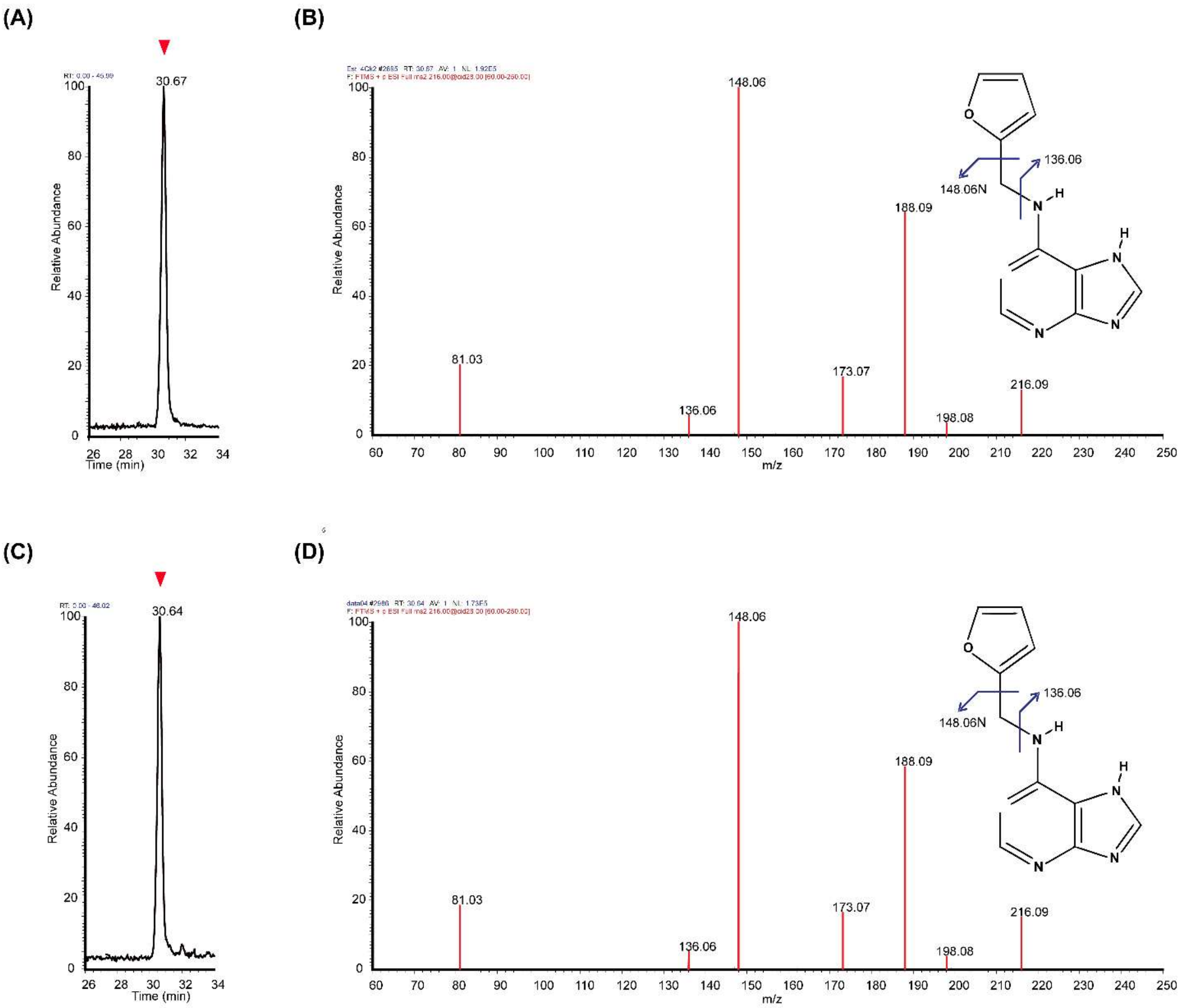

Figure S5. Chromatograms and fragmentation pattern obtained for Kinetin (K) by LC-MS/MS. (**A** and **B**) Chromatograms and fragmentation pattern for analyte. (**C** and **D**) Chromatograms and fragmentation pattern obtained from pre-inducted leaf samples. Red arrowheads in chromatograms indicate the retention time for K.

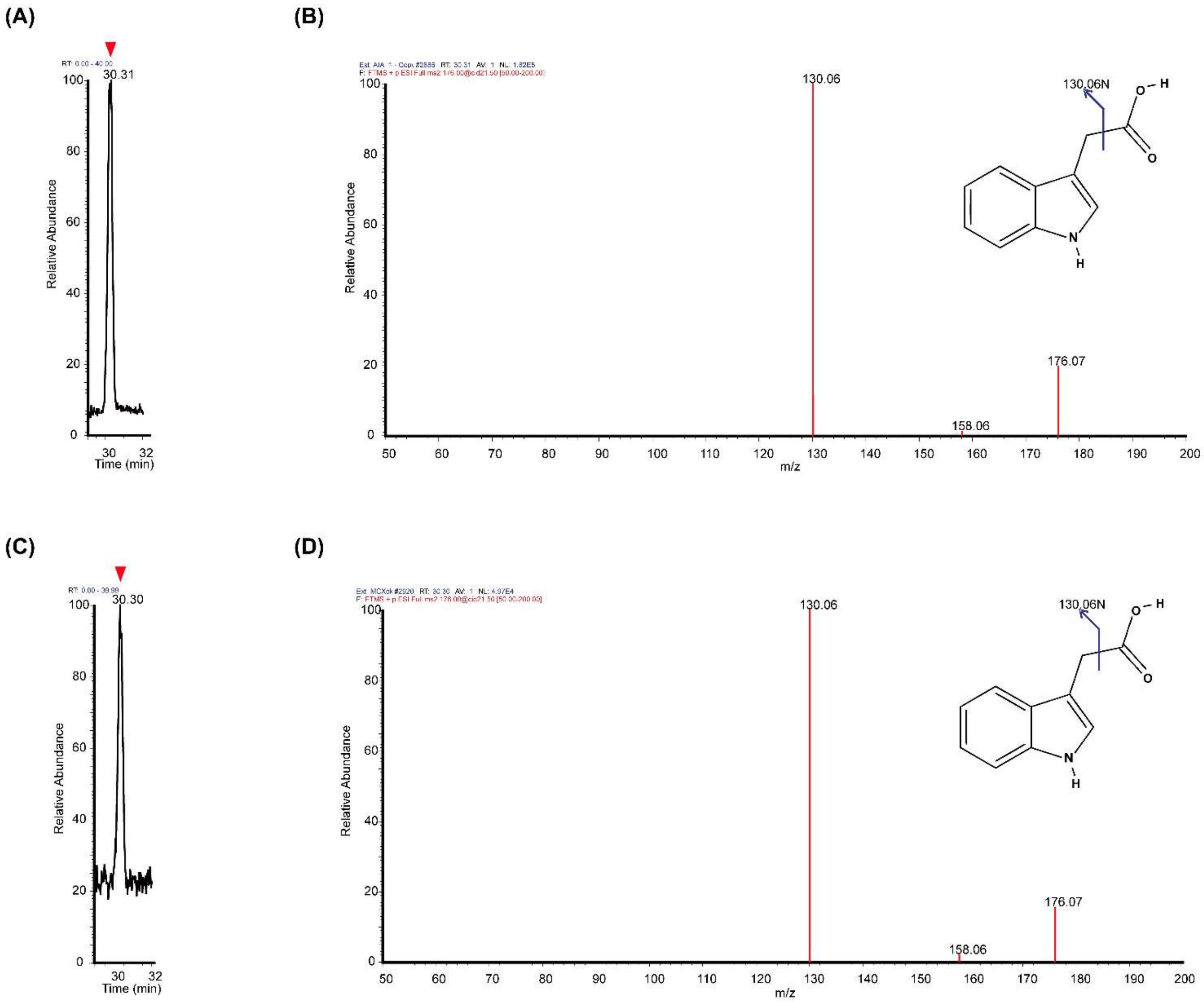

Figure S6. Chromatograms and fragmentation pattern obtained for Indole-3-Acetic Acid (IAA) by LC-MS/MS. (**A** and **B**) Chromatograms and fragmentation pattern for analyte. (**C** and **D**) Chromatograms and fragmentation pattern obtained from pre-inducted leaf samples. Red arrowheads in chromatograms indicate the retention time for IAA.

Table S1. Gene-specific primer sequences used for Quantitative Real-time RT-qPCR amplification.

Gene	Sequences of the primers
CcIPT1	F-5′-GTCCAACTCCTTCATCTACTCC-3′
	R-5′-GTACACGTGTTCTCCTTGACAG-3′
CcIPT5	F-5′-GTACAGGGTTACCACTTGTTCC-3′
	R-5′-GTGGATGTTTCTGGTTCTCC-3′
CcCKK3	F-5′-CCTCTACTGCATGGAAGTTGTC-3′
	R-5′-GTACGACAGCAGACATTCTCTC-3′
CcCKK9	F-5′-GTCGAGTTCCTGGATAGAGTTC-3′
	R-5′-GTGGATGTTTCTGGTTCTCC-3′
CcARR2	F-5′-CAGCAGATGTCTACAGGAAGG-3′
	R-5′-GAGACCACAGTTAGGTCATTGC-3′
CcUBIQUITIN	F-5′-TTCGTCAAGACCCTCACC-3′
	R-5′-TCAAACGGAGAACCAAGTG-3′

Table S2. Overview of tandem mass spectral and UHPLC features for the PGRs detected.

PGR[a]	Parent ion formula	Parent MH$^+$	Fragments	CID[a] (V)	Analyte RT[b] (min)	Plant RT[b] time (min)
trans-Zeatin (tZ)	$C_{10}H_{13}N_5O$	220.12	202.11, 148.06, 136.06	25.5	18.32 ± 0.07	18.31 ± 0.06
Zeatin riboside (ZR)	$C_{15}H_{21}N_5O_5$	352.16	220.12, 202.11, 148.06, 136.06	23.0	29.93 ± 0.06	29.93 ± 0.07
Kinetin (K)	$C_{10}H_9N_5O$	216.09	188.09, 173.07, 148.06, 136.06, 81.03	28.0	30.67 ± 0.07	30.64 ± 0.06
Indole-3-acetic acid (IAA)	$C_{10}H_9NO_2$	176.07	130.06	21.5	30.31 ± 0.09	30.30 ± 0.08

a, Collision-induced dissociation; b, indicates the retention time (RT) in minutes (min). To confirm the identity of recorded PGRs, UHPLC coupled to mass spectrometry was used. In the MS/MS analysis, the collision of the protonated ion of tZ and ZR showed the characteristics of ions that matched the adenine with part of (m/z 148.08) or without (m/z 136.06) the isoprenoid side chain (Table S1). The ZR MS/MS spectra displayed the neutral loss of m/z 132 corresponding to ribose, thus resulting in the ion m/z 220.12. In the K spectra, the protonated ion collision showed that the characteristics of ions matched the adenine with part of (m/z 148.08) or without (m/z 136.06) the aromatic side chain. The Aux IAA showed the characteristic fragment m/z 130.06. When fragmentation patterns from analytes and *C. canephora* extracts were compared, similar MS/MS spectra were found at the same RT (Figures S3-S6).
